# Mesenchymal stem cells and JIA

**DOI:** 10.1186/1546-0096-9-S1-P65

**Published:** 2011-09-14

**Authors:** JF Swart, S de Roock, F Hofhuis, W Kuis, BJ Prakken, AC Martens, I Slaper-Cortenbach, NM Wulffraat

**Affiliations:** 1 Department of Pediatric Immunology, Wilhelmina Children’s Hospital/UMCU, Utrecht, Netherlands; 2 Department of Immunology, UMC Utrecht, Utrecht, Netherlands

## Background

Mesenchymal stem cells (MSC) are adult stem cells present in bone marrow (BM), fat and in many other tissues. MSC have strong immunosuppressive qualities in vitro. The immunosuppression seems to work both by paracrine action and cell-cell contact. MSC inhibit NK-cells, B-cells, dendritic cells, Th1-cells and activate regulatory T-cells. The presence of floating single MSC in human synovial fluid (SF) is a normal phenomenon and the number of MSC in SF is much lower in RA than in osteoarthritis (OA). SF in RA might be impaired in recruiting MSC to the joint and the proliferation potential of MSC in RA patients could be suppressed by the synovitis or be exhausted as suggested by the decreased telomere length. Administration of additional MSC might therefore have great potential in the treatment of inflammatory arthritis as we and others have shown in different experimental arthritis models.

## Aim

To evaluate the presence and number of MSC in SF of JIA patients and to see if addition of healthy MSC was capable of immunosuppression of the synovial T-cells in JIA patients.

## Methods

We used the criteria of the ISCT [Horwitz EM etal. Cytotherapy. 2005;7(5):393-5.] to culture the MSC and used CFSE staining for lymphocytes and our standard setting for suppression with regulatory T-cells as described [Vercoulen Y etal. Plos One 2009; 4(9)].

## Results

**Figure 1:**
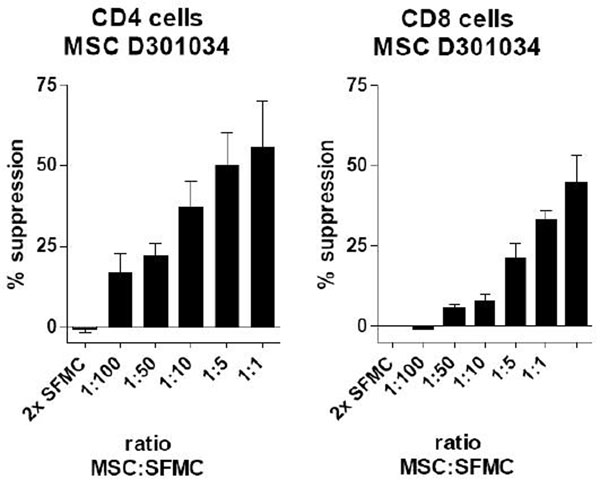


In JIA patients we find around 5-10 x10^6^ cells per milliliter SF with 1.2-2.4 x10^6^ CD4+ cells and 1.6-3.2 x10^6^ CD8+ cells per ml. In the SF of arthritic knees from 14 JIA patients we found mesenchymal stem cells at a low rate: a median of 4 CFU-F (with a maximum of 81 CFU-F) per million SF cells [normal healthy SF contains 250 CFU-F per 1 million SF cells]. We found that adding allogeneic healthy BM derived MSC to anti-CD3 stimulated SF cells of JIA patients showed dose dependent suppression of SF CD4+ and CD8+ T cell proliferation (Fig 1).

## Conclusion

A low number of CFU-F were found in the SF of JIA patients. The addition of allogeneic MSC to SF of JIA patients suppressed the synovial CD4+ and CD8+ T cell proliferation. A ratio of 1 MSC per 5 CD4+ or 5 CD8+ cells already yielded good results.

